# A case of thoracic central venous obstruction treated by the innominate-to-right-atrial bypass grafting technique under extracorporeal circulation

**DOI:** 10.1093/jscr/rjae050

**Published:** 2024-02-23

**Authors:** Jianfeng Chen

**Affiliations:** Department of Anesthesiology, West China Hospital, Sichuan University, No. 37 Guoxue Alley, Wuhou District, Chengdu, Sichuan 610041, China

**Keywords:** thoracic central venous obstruction, central venous access, cuffed dialysis catheter, innominate vein, right atrium

## Abstract

A 46-year-old woman with stage 5 chronic kidney disease was unable to undergo hemodialysis treatment due to thoracic central venous obstruction (TCVO) and blockage of the tunneled cuffed catheter. This patient also presented with symptoms of TCVO. When percutaneous procedure was not possible, we resolved the obstruction with the innominate-to-right-atrial bypass grafting technique under extracorporeal circulation. There are few reports on this surgical approach. In terms of patient prognosis, this may be an effective solution to this problem.

## Introduction

As the number of patients with long-term hemodialysis needs increases, the number of patients developing thoracic central venous obstruction (TCVO) continues to rise. It was found to be related to chronic irritation of the vascular endothelium by patient-placed catheters [1]and the high-flow status during dialysis [2]. Repeated stimulation of the intravascular catheter can lead to TCVO by causing neointimal hyperplasia [3], wall thrombosis and fibrosis [4], and formation of fibrin sheaths at and around the tip of the catheter [5]. This not only causes oedema in the patient’s ipsilateral arm and other areas [6], but may also leads to superior vena cava syndrome [7]. For patients who need long-term hemodialysis, this problem can be fatal if not solved quickly. This case study discusses the use of surgical methods to solve TCVO when percutaneous procedure is not feasible.

## Case report

A 46-year-old woman was diagnosed with chronic kidney disease stages 5 over 20 years ago. She has undergone three times kidney transplantation but still requires hemodialysis three times a week. During this period, the patient’s upper extremity arteriovenous fistulas repeatedly lost function and she was performed multiple arteriovenous fistula surgeries and dialysis catheter placement. In 2019, a tunneled cuffed catheter was placed in the right internal jugular vein for hemodialysis, but the patient continued to experience repeated catheter dysfunction and underwent multiple tunneled cuffed catheter replacements. According to the digital subtraction angiography (DSA) examination, it was found that the right innominate vein, brachiocephalic vein, and superior vena cava gradually became stenosis. A total of 1 week ago, the tunneled cuffed catheter in the patient’s right internal jugular vein showed a decrease in blood flow during dialysis again (˂180 ml/min), and she was scheduled to be admitted to the hospital again for tunneled cuffed catheter replacement. It was found that the tip of the patient’s tunneled cuffed catheter was located in the right atrium, with thrombus attached, and thrombosis was found in the right lower lobe and inferior vena cava according to DSA. The right internal jugular vein to right brachiocephalic vein, junction of the left and right brachiocephalic vein to superior vena cava were narrow. After evaluation, it was impossible to replace the tunneled cuffed catheter under percutaneous procedure. The patient’s central venous return was obstructed, and symptoms of TCVO have appeared. The thrombus in the right atrium may also detach again and cause pulmonary embolism, and hemodialysis cannot be performed normally.

**Figure 1:**
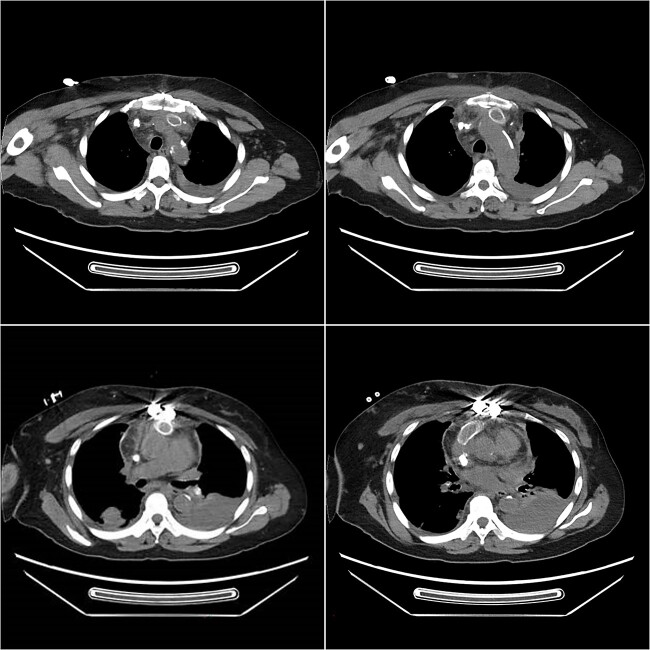
Postoperative computed tomography angiography (CTA) showed no contrast agent leakage in the artificial blood vessel.

**Figure 2:**
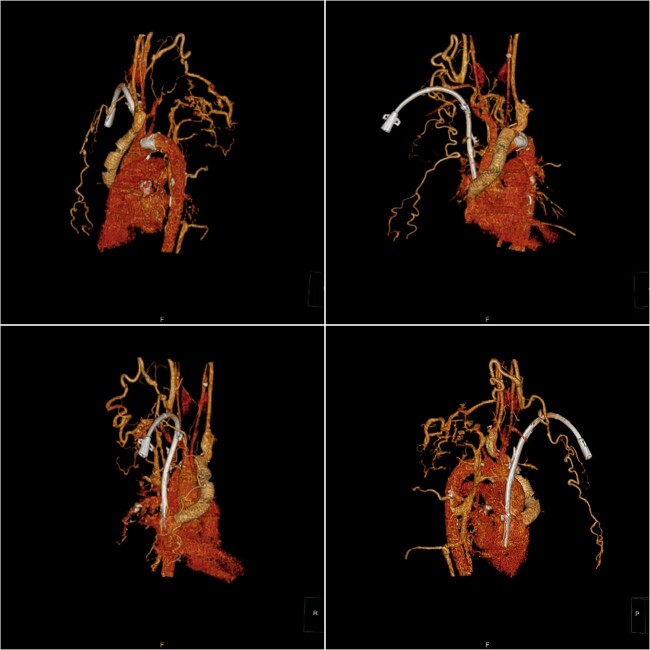
Postoperative computed tomography (CT) three-dimensional reconstruction shows the artificial blood vessel and tunneled cuffed catheter deformation.

After a cardiac surgery consultation, the innominate-to-right-atrial bypass grafting technique under extracorporeal circulation and right atrial thrombus removal was planned. After anesthesia induction, the patient underwent central venous catheter placement in the left femoral vein as a route for anesthetic and vasoactive drugs. After the patient was fully heparinized, the sternum was opened for aortic cannulation and inferior vena cava drainage to establish extracorporeal circulation. During the operation, the right atrium was opened and a large calcified thrombus attached to the tunneled cuffed catheter was removed. Subsequently, the narrowed part of the unnamed vein was excised, and an artificial blood vessel (Gore R14030030L14mm^*^30 cm^*^30 cm) was anastomosed to the proximal end. After excising part of the right atrial appendage, the distal end of the artificial blood vessel was anastomosed to it, forming an innominate vein-to-right atrium artificial blood circulation (Fig. 1). The tracheal catheter was successfully removed after the operation. The patient currently has no symptoms such as arm swelling. The artificial blood vessel is unobstructed (Fig. 2), and normal dialysis is performed using the tunneled cuffed catheter.

## Discussion

For patients requiring long-term hemodialysis, the treatment of TCVO needs not only to relieve the symptoms of venous obstruction, but also to provide the patient with stable access to dialysis. In this case, a large of thrombi had formed around the patient’s tunneled cuffed catheter and in the right atrium, occlusion of multiple vessels had occurred, making it difficult to perform interventional procedures such as percutaneous transluminal angioplasty [8] and stent placement [9]. The use of vascular bypass grafting may be an effective solution to TCVO [10].

Duncan [11] and others firstly proposed a surgical method of connecting the right atrium bypassing the obstructed central vein with an autologous or artificial vessel, which theoretically allows the blood from the central vein to return directly to the right atrium bypassing the site of the obstruction and relieves the various symptoms caused by central venous hypertension in patients. To date, there have been only a small number of studies on this surgical procedure [12]，and even fewer studies on the maintenance of access in dialysis patients after this procedure.

In this case, we removed the thrombus in the patient’s right atrium intraoperatively, which prevented the continuation of pulmonary embolism to a certain extent and ensured the smoothness of dialysis access. Due to the patient’s central venous obstruction, femoral vein cannulation was performed after induction of anesthesia as an intraoperative access for anesthetic drugs and vasoactive drugs. Adequate peripheral vascular tone was maintained to ensure the stability of the circulation during the operation. Choosing the right artificial vessel to prevent collapse; optimizing flow rate to enable prolonged use of artificial vessel [13]; choosing the right position for graft anastomosis to prevent kinking; these may be the reasons for the success of the surgery. In the postoperative period active prevention of graft infection and regular dialysis to prevent heart failure are particularly important for the patient’s prognosis. Since patients with end-stage renal disease often have abnormal platelet function, the need for postoperative anticoagulation or antiplatelet therapy is still controversial.

In this case, the patient was reviewed postoperatively. The patency of the artificial vessel was confirmed, the pressure of the tunneled cuffed catheter was normal, and the TCVO symptoms disappeared. It suggests that the innominate-to-right-atrial bypass grafting technique may be an important surgical procedure for patients with TCVO for whom percutaneous intervention is not possible. Further relevant studies in large samples are still needed to confirm the effectiveness of this treatment.
